# Nrf2 as a Potential Mediator of Cardiovascular Risk in Metabolic Diseases

**DOI:** 10.3389/fphar.2019.00382

**Published:** 2019-04-12

**Authors:** Rafael M. da Costa, Daniel Rodrigues, Camila A. Pereira, Josiane F. Silva, Juliano V. Alves, Núbia S. Lobato, Rita C. Tostes

**Affiliations:** ^1^Department of Pharmacology, Ribeirão Preto Medical School, University of São Paulo, São Paulo, Brazil; ^2^Special Academic Unit of Health Sciences, Federal University of Goiás, Jataí, Brazil

**Keywords:** Nrf2, oxidative stress, cardiovascular risk, metabolic diseases, therapeutic target

## Abstract

Free radicals act as secondary messengers, modulating a number of important biological processes, including gene expression, ion mobilization in transport systems, protein interactions and enzymatic functions, cell growth, cell cycle, redox homeostasis, among others. In the cardiovascular system, the physiological generation of free radicals ensures the integrity and function of cardiomyocytes, endothelial cells, and adjacent smooth muscle cells. In physiological conditions, there is a balance between free radicals generation and the activity of enzymatic and non-enzymatic antioxidant systems. Redox imbalance, caused by increased free radical’s production and/or reduced antioxidant defense, plays an important role in the development of cardiovascular diseases, contributing to cardiac hypertrophy and heart failure, endothelial dysfunction, hypertrophy and hypercontractility of vascular smooth muscle. Excessive production of oxidizing agents in detriment of antioxidant defenses in the cardiovascular system has been described in obesity, diabetes mellitus, hypertension, and atherosclerosis. The transcription factor Nrf2 (nuclear factor erythroid 2–related factor 2), a major regulator of antioxidant and cellular protective genes, is primarily activated in response to oxidative stress. Under physiological conditions, Nrf2 is constitutively expressed in the cytoplasm of cells and is usually associated with Keap-1, a repressor protein. This association maintains low levels of free Nrf2. Stressors, such as free radicals, favor the translocation of Nrf2 to the cell nucleus. The accumulation of nuclear Nrf2 allows the binding of this protein to the antioxidant response element of genes that code antioxidant proteins. Although little information on the role of Nrf2 in the cardiovascular system is available, growing evidence indicates that decreased Nrf2 activity contributes to oxidative stress, favoring the pathophysiology of cardiovascular disorders found in obesity, diabetes mellitus, and atherosclerosis. The present mini-review will provide a comprehensive overview of the role of Nrf2 as a contributing factor to cardiovascular risk in metabolic diseases.

## Overview of Reactive Oxygen Species and Cardiovascular Function

Although reactive oxygen species (ROS) were initially presumed to cause cell damage, they are now recognized as important molecules that regulate many cell signaling and biological processes, such as induction of defense genes, activation of transcription factors, phosphorylation of kinases, and mobilization of ions in transport systems (reviewed in Droge, 2002; Touyz and Briones, 2011; Brown and Griendling, 2015). In the cardiovascular system, ROS generation is important to maintain endothelial and vascular smooth muscle cells (VSMCs) function, including vascular tone control, inflammation-related responses, cell growth and proliferation, modulation of extracellular matrix production, apoptosis and angiogenesis (Siti et al., 2015; reviewed in Galley and Straub, 2017). Alterations of the balance between cellular ROS production and the capacity to rapidly detoxify reactive intermediates play an important role in the development of risk factors for cardiovascular diseases (reviewed in Harrison et al., 2003 and Touyz et al., 2018). Such events are observed in patients with essential hypertension (Redón et al., 2003) and various experimental models of arterial hypertension, such as spontaneously hypertensive rats (SHR) that exhibit increased levels of vascular superoxide anion and vascular smooth muscle hypercontractility (Paravicini et al., 2004). The same occurs in obesity and experimental models of diabetes, in which insulin resistance and hyperglycemia culminate in increased NAD(P)H oxidase enzyme activity and endothelial nitric oxide synthase (eNOS) uncoupling, thereby contributing to increased ROS generation and impaired vascular function (Youn et al., 2012; da Costa et al., 2017; Neves et al., 2018).

Vascular cells generate ROS in response to several stimuli, including cytokines, angiotensin II, endothelin-1, aldosterone and platelet-derived growth factor (PDGF) (reviewed in Thannickal and Fanburg, 2000). ROS signaling in endothelial cells and VSMCs involves alterations in the intracellular redox state and oxidative modification of regulatory and contractile proteins (da Costa et al., 2017). The oxidative modification of these redox-sensitive proteins alters their conformation, stability, activity and/or ability to interact with other proteins, resulting in modulation of vascular function. Redox-sensitive proteins include proteins involved in calcium handling as well as contractile proteins, proteins involved in various signaling and transcriptional activities. Redox modulation of calcium handling proteins directly affects cardiac and vascular contraction by altering intracellular calcium levels. Examples of redox-sensitive calcium handling proteins are calcium-calmodulin kinase II (CaMKII), the ryanodine receptor on the sarcoplasmic reticulum, sarcoplasmic reticulum ATPase (SERCA), and phospholamban (reviewed in Burgoyne et al., 2012 and Steinberg, 2013). Protein kinases and phosphatases are also affected by direct redox modification, resulting in modulation of various signal transduction pathways in cardiac and vascular cells, including altered modulation of calcium sensitivity, phosphorylation of the myofilaments and receptor tyrosine kinase signaling (reviewed in Knock and Ward, 2011 and Steinberg, 2013).

The long-term effects of ROS in cardiovascular function depend on the balance between signals promoting proliferation or growth inhibition and/or cell death. This dual role is evidenced by the findings that endothelial apoptosis initiated by tumor necrosis factor-alpha (TNF-α) is attenuated by ROS scavenging (Xia et al., 2006) and that, during ischemia-reperfusion injury, ROS trigger apoptosis, whereas ROS generated during ischemic preconditioning prevent apoptosis (Maulik et al., 1999; Hattori et al., 2001; reviewed in Becker, 2004). In vascular cells, ROS can alter this balance leading to either excessive angiogenesis or loss of endothelial cells (reviewed in Papaharalambus and Griendling, 2007).

To contain ROS generation, there are antioxidant defenses that prevent or neutralize the generation of highly reactive compounds in biological systems. Among the major intracellular antioxidant enzymes, superoxide dismutase, catalase, and glutathione peroxidase are major players. In cooperative action, these enzymes catalyze ROS in molecular oxygen and water (reviewed in Rodriguez and Redman, 2005). Other agents, such as ascorbic acid (reviewed in May and Harrison, 2013) and phenolic acids (reviewed in Santhakumar et al., 2014), which do not present enlightened mechanisms of action, also contribute to redox balance.

Although the exact mechanisms underlying cardiovascular oxidative stress during obesity, diabetes, and atherosclerosis still remain unclear, the discovery of mechanisms and subsequent new pharmacological interventions related to the disruption of the antioxidant system has become of great interest to identify key mediators that will potentially instigate and exacerbate these cardiovascular complications.

## Nrf2 Activity and Regulation

Several cell types throughout evolution have developed adaptive programs to counteract oxidative stress, and the role of the nuclear factor erythroid 2-related factor-2 (Nrf2) in these programs has been a subject of recent interest. Nrf2 is a basic leucine zipper region (bZip) transcription factor of the Cap n’Collar family, with approximately 589 amino acid residues and seven conserved domains (Neh1 – Neh7) (reviewed in Kansanen et al., 2013). When the levels of ROS and electrophiles become greater than the cell’s ability to detoxify them, a series of events results in Nrf2 activation. Activated Nrf2 heterodimerizes with Jun and small musculoaponeurotic fibrosarcoma (sMaf) proteins, translocates to the nucleus and binds to the antioxidant response element (ARE) or the electrophile-response element (EpRE) in the promoter region of Nrf2 target genes. This results in the coordinated expression and activation of antioxidant, antiapoptotic, metabolic, and detoxification proteins. Among the proteins with antioxidant activity regulated by Nrf2 are superoxide dismutase (SOD), catalase (CAT), heme-oxygenase 1 (HO-1), glutathione peroxidase 1 (GPx-1) and NAD(P)H: quinone oxidoreductase 1 (reviewed in Tebay et al., 2015).

The conserved Nrf2 domains are crucial for their self-regulation and activity. In this context, the Neh-1 domain is responsible for the binding of Nrf2 to DNA through dimerization with the low molecular weight sMaf proteins of type F, G, and K (sMafF, sMafG, and sMafK), favored by the bZip domain, and by recognition of the ARE (Mohler et al., 1991; Oyake et al., 1996; reviewed in Motohashi et al., 2002). The Neh-2 domain, rich in lysines, is responsible for binding of Nrf2 to its cytoplasmic repressor protein Keap-1. This domain also has high and low affinity regions for Keap-1 binding, the ETGE and DLG motifs, respectively (Itoh et al., 1999; Kobayashi et al., 2002). The Neh-3 domain, located in the carboxy-terminal portion of Nrf2, is sumoylated. This post-translational modification is important for Nrf2 transactivation. In addition to Neh-3, the Neh-4, and Neh-5 domains, when acetylated or oxidized, in oxidative stress conditions, are responsible for the transactivation of Nrf2 (Katoh et al., 2001; Nioi et al., 2005). The Neh-6 and Neh-7 domains are responsible for the degradation of Nrf2, independently of the regulation by Keap-1 (McMahon et al., 2004; Wang et al., 2013).

In physiological conditions, when there is a balance between oxidant species and the adequate activity of antioxidant proteins, Nrf2 is suppressed in the cytoplasm. This suppression occurs adjacent to the cellular cytoskeleton by the interaction of Nrf2 with Keap-1 and Cullin3, a protein of the E3 ligase family (reviewed in Canning et al., 2015). The formation of the Nrf2-Keap-1-Cullin3 complex becomes a signal for the ubiquitination and proteasome degradation of Nrf2, maintaining its basal levels along with the genes regulated by this transcription factor (Furukawa and Xiong, 2005; reviewed in Canning et al., 2015).

Under oxidative or electrophilic stress conditions, ROS promote the breakdown of the Nrf2-Keap1-Cullin3 complex, allowing Nrf2 to translocate to the nucleus and activate the expression of antioxidant proteins. The ARE is normally suppressed by a heterodimer formed by the sMaf and Bach-1 proteins, preventing Nrf2 heterodimerization and binding to the ARE (reviewed in Tebay et al., 2015 and Tong et al., 2015). However, under conditions of oxidative stress, there is an accumulation of heme groups in the cytosol, a frequent substrate for ROS formation. In this condition, Bach-1 undergoes dissociation of sMaf proteins and migrates to the cytosol to act on the heme group metabolism, allowing Nrf2 to dimerize with the sMaf protein and initiate gene transcription (reviewed in Ryter and Choi, 2005 and Tebay et al., 2015).

Among the proteins that participate in Nrf2 regulation and activity, Keap-1 and Bach-1 stand out from the others due to their direct role in redox balance and cardiovascular homeostasis (Lopes et al., 2015). Of importance, the negative regulation of Nrf2 by Keap-1 and Bach-1 raises the possibility of new therapeutic targets to prevent oxidative stress-associated cardiac hypertrophy and vascular dysfunction (Abed et al., 2015; Jiao et al., 2018).

## Nrf2 and Obesity

The mechanisms accompanying the progression of obesity and its cardiovascular comorbidities have been the focus of considerable research over the last 30 years. As the mechanistic insights clarify, it is evident that the expansion of visceral adipose tissue plays a pivotal role in the pathophysiology of obesity. Increased ROS generation by the adipocytes, which, in turn, increases expression and secretion of inflammatory adipokines, causes dramatic consequences for the regulation of energy homeostasis and vascular function (Keaney et al., 2003; reviewed in DeMarco et al., 2010 and Otani, 2011). This is a common combination associated with individual cardiovascular disease risk (Furukawa et al., 2004; reviewed in Sowers et al., 2011).

Recent evidence shows that Nrf2 is involved in the control of energy metabolism and might be a promising therapeutic target for obesity. The underlying mechanism of Nrf2 in obesity has been investigated using various experimental approaches, including Nrf2 gene deletion, Nrf2 pharmacological activators and Nrf2 gene overexpression, but only few of these approaches were clinically tested. Specific pharmacological activators of Nrf2, including epigallocatechin 3-gallate, oltipraz, sulforaphane, curcumin and 1-[2-cyano-3, 12-dioxooleana-1, 9(11)-dien-28-oyl] imidazole (CDDO-Im), induce the expression and activity of Nrf2 both *in vitro* and *in vivo* (Shin et al., 2009; Yu et al., 2011; Xu et al., 2012; Gao et al., 2013). The epigallocatechin 3-gallate-induced activation of Nrf2 in liver and adipose tissue of obese mice improves lipidemic control, decreases oxidative products generation, and reduces body mass, insulin and glucose levels (Sampath et al., 2017). Interestingly, knockout mice for the Keap-1 protein exhibit similar features, including suppression of high-fat diet-induced obesity and decreased deposition of lipids and cholesterol in the liver (Slocum et al., 2016). Of importance, improvement of metabolic profile is closely associated with improved cardiovascular function (da Costa et al., 2016; Neves et al., 2018).

A recent study demonstrated that the Nrf2 activator sulforaphane, during revascularization procedures in metabolically compromised individuals, has the potential to suppress the progression of intimal hyperplasia. In addition, Nrf2 activation attenuates leptin-induced proliferation of VSMCs in the diet-induced obesity scenario (Shawky et al., 2016).

Natural compounds are also promising elements in Nrf2 activation during obesity. As an example, Zeng et al. (2015) observed that curcumin, a natural Nrf2 activator, suppresses oxidative stress, inflammation and hypertrophy induced by treatment of cardiac cells with free fatty acids. Similarly, high-fat diet induced oxidative stress, inflammation, fibrosis, hypertrophy and tissue remodeling are attenuated by curcumin treatment. These benefits are closely associated with increased Nrf2 expression and activity, as well as reduced ROS generation (Zeng et al., 2015).

Deletion of the Nrf2 gene is expected to increase ROS generation and to aggravate the phenotypes of obesity. Nrf2 knockout mice show increased ROS generation, deposition of fatty acids in the liver and increased expression of genes related to the synthesis of lipids and cholesterol (Tanaka et al., 2008). Consistent with these findings, our group showed that obesity in mice favors vascular oxidative stress by increasing the expression of the downregulatory proteins of Nrf2, Keap-1 and Bach-1 (Costa et al., 2017).

Nrf2 in adipose tissue function and metabolic syndrome has also been examined. Xue et al. (2013) determined the role of Nrf2 in the development of obesity and associated metabolic disorders, using Nrf2-knockout mice on a leptin-deficient ob/ob background, a model with an extremely positive energy balance. In obese mice, ablation of Nrf2, globally or specifically in adipocytes, reduces white adipose tissue mass. However, Nrf2 deletion results in even more severe metabolic syndrome with aggravated insulin resistance, hyperglycemia, and hypertriglyceridemia. In addition, when compared to wild-type mice, the white adipose tissue of obese mice expresses substantially higher levels of many genes related to antioxidant response, inflammation, adipogenesis, lipogenesis, glucose uptake, and lipid transport (Xue et al., 2013). These findings support a role for Nrf2 in regulating adipose tissue development and function, insulin sensitivity, glucose and lipid homeostasis.

In contrast to the above-mentioned study, Nrf2 knockout mice treated with a high-fat diet tend to gain less body mass and display increased insulin sensitivity and glucose tolerance. In addition, they do not exhibit increased glucose, cholesterol, or plasma triglycerides. Of importance, the altered metabolic phenotype of Nrf2- knockout mice on high-fat diet is associated with higher expression and abundance of fibroblast growth factor 21 (FGF21), a novel hormone that regulates energy metabolism, glucose tolerance and adipose tissue expansion (Chartoumpekis et al., 2011).

The complex roles of Nrf2 in adipogenesis and adipose tissue functions were recently examined by adipose tissue-specific ablation of Nrf2 in mice. This condition is associated with a transient delayed increase of body weight in high-fat diet-fed mice. However, the benefit is eventually suppressed after prolonged feeding. The phenotypic changes induced by adipose tissue-specific ablation of Nrf2 also extend to the whole-body level, reducing blood glucose and altering the expression of genes involved in glucose, lipid and energy metabolism (Zhang et al., 2016). These findings are consistent with those of previous studies using Nrf2 knockout mice (Pi et al., 2010; Xu J. et al., 2015) and provide additional mechanistic insights into the role of Nrf2 as an important mediator of glucose, lipid and energy metabolism. To date there are no studies showing direct effects of Nrf2 deletion on cardiovascular function.

The apparent contradictory role of Nrf2 protecting mice from obesity and insulin resistance in conditions of Nrf2 deficiency in comparison to Nrf2 pharmacological activation, may be explained by the observations that Nrf2 deficiency leads to a mild increase in the levels of ROS, which stimulate the antioxidant system in a manner similar to the Nrf2 activators (reviewed in Bocci et al., 2014). Another possible explanation is that Nrf2 activators also regulate non-Nrf2 signaling pathways to modulate glucose and lipid metabolism. The same is true for Keap-1, the Nrf2 repressor protein, which may also have Nrf2-independent effects on transcription factors and, consequently, on metabolic homeostasis (Huang et al., 2012). Controversial results on the role of Nrf2 in obesity may be linked to differences in the pathophysiological characteristics of obesity (such as diet content or time under obesity conditions), as well as specific genetic characteristics. Table 1 summarizes the contribution of Nrf2 signaling in different obesity conditions. Further studies are required to explore this apparent discrepancy on the role of Nrf2 in obesity.

**Table 1:** Nrf2 signaling and actions in obesity and atherosclerosis animals models.

Genotype strain mice	Metabolic and body parameters	Atherosclerotic plaque	Diet / feeding time period	Effect on Nrf2 and target genes	References
Male C57BL/6J	HFD vs. LFD	Not assessed	LFD (10% calories from fat) or HFD (45% calories from fat), and HFD + E-25 or HFD + E-75 for 17 weeks.	HFD vs. LFD	Sampath et al. (2017)
	↓ glycaemia and body weight.			↔ Nrf2 nuclear fraction in the liver.	
	HFD + E-75 vs. LFD			HFD + E-75 vs. LFD	
	↔ glycaemia and body weight.			↑ Nrf2 nuclear fraction and HO-1 protein expression.	

Male C57BL/6J WT and Keap1-hypo	HFD Keap1-hypo vs. HFD WT	Not assessed	SD (10 kcal % fat) or HFD (60 kcal % fat) for 90 days.	Keap1-hypo vs. WT	Slocum et al. (2016)
	↓ glycaemia, hepatic triglyceride and body weight.			↑ NQO-1 mRNA.	

Male C57BL/6J	HFHS+SFN vs. HFHS	HFHS+SFN vs. HFHS	SD (10% calories from fat and 72% from carbohydrate) or HFHS diet (40% calories from fat, 42% from carbohydrate and 0.15% w/w cholesterol), and HFHS + SFN for 8 weeks.	SFN induces Nrf2 activation.	Shawky et al. (2016)
	↓ glycaemia, weight gain, plasma leptin, plasma insulin, cLDL and triglycerides.	↓ neointima formation in the injured femoral artery.			

Male C57BL/6J	HFD curcumin vs. HFD	Not assessed	SD or HFD, and HFD + curcumin treatment for 8 weeks.	HFD curcumin vs. HFD	Zeng et al. (2015)
	↔ triglycerides, LDL, cholesterol total and body weight.			↑ Nrf2, HO-1 and NQO-1 gene and protein expression in the myocardium.	

Male C57BL/6J WT; Nrf2^+/+^:ob/ob; Nrf2^-/-^:ob/ob and Adipocyte-specific Nrf2-KO	Nrf2^-/-^:ob/ob vs. Nrf2^+/+^:ob/ob	Not assessed	SD for 11 weeks.	WT vs. Nrf2^+/+^:ob/ob	Xue et al. (2013)
	↓ weight gain and white adipose tissue;			↑ HO-1 and NQO-1 mRNA.	
	↑ insulin resistance, triglycerides, glycaemia.				
	Adipocyte-specific Nrf2-KO and Nrf2^-/-^:ob/ob mice have a similar phenotype.				

Male C57BL/6J WT and Nrf2^-/-^	Nrf2-/- HFD vs. HFD WT	Not assessed	SD (10 kcal % fat) or HFD (60 kcal % fat) for 180 days.	HFD WT vs. SD WT	Chartoumpekis et al. (2011)
	↓ weight gain, basal glucose, insulin resistance, leptin.			↑ Nrf2 mRNA.	
	↑ triglycerides.				

Male adipose-specific Nrf2-KO (NK) and Nrf2 control (NC)	NK HFD vs. NC HFD	Not assessed	SD (5.55% kcal soybean oil and 4.44% kcal) or HFD (5.55% kcal soybean oil and 54.35% kcal) for 14 weeks.	NK mice has a reduction in adipose tissue Nrf2 expression	Zhang et al. (2016)
	↓ weight gain, basal glucose.				
	↔ cholesterol, leptin, free fatty acid.				

Male C57BL/6J WT and Nrf2^-/-^	Nrf2^-/-^ HFD vs. WT HFD, 12 weeks	Not assessed	Regular diet (11% fat) or HFD (41% fat) for 4, 8, and 12 weeks.	Not assessed	Pi et al. (2010)
	↓ weight gain, adipose tissue.				

Male Lep^*ob/ob*^ (OB) and Nrf2/Lep^*ob/ob*^ (OB-Nrf2 KO)	OB-Nrf2 KO vs. OB, 8 weeks	Not assessed	SD for 4, 8, and 12 weeks.	Not assessed	Xu J. et al., 2015
	↓ body weight, adipose tissue, glucose tolerance.				
	↑ VLDL/triglycerides hepatic secretion, triglycerides, cholesterol.				
	↔ not-fasting glucose.				

Male C57BL/6J WT and Nrf2^-/-^	Not assessed	Not assessed	SD and Carotid artery treatment with oxPAPC for 6 h and 24 h.	WT + oxPAPC vs. WT	Jyrkkänen et al. (2008)
				↑ HO-1 and NQO-1 expression.	

Female	↔ cholesterol.	↑ Atherosclerotic lesion formation.	Western Diet (1.25% cholesterol and 21% fat) for 8 weeks.	Not assessed	Yet et al. (2003)
HO-1^-/-^:ApoE^-/-^ and HO-1^+/+^:ApoE^+/+^					

Male Nrf2^-/-^:ApoE^-/-^ and Nrf2^+/+^:ApoE^-/-^	↓ cholesterol, VLDL in Nrf2^-/-^:ApoE^-/-^ irradiated.	↔ Atherosclerotic lesion at 3 and 5 weeks.	HFD (1.25% cholesterol) for 3, 5, and 12 weeks.	↓ HO-1 expression in Nrf2^+/+^: ApoE^-/-^ with HFD at 12 weeks.	Harada et al. (2012)
		↓ atherosclerotic plaque at 12 weeks in Nrf2^-/-^: ApoE^-/-^.			

Male LDLr^-/-^ and LDLr^-/-^ transplanted with Nrf2^-/-^ BM (Nrf2^+/+^ BM)	↔ cholesterol, triglycerides.	↑ Lesion, necrotic cord.	SD or HFD.	HFD-fed Nrf2^-/-^ vs. Nrf2^+/+^ BM mice	Collins et al. (2012)
				↓ NQO-1, catalase, Gpx-1.	

Male C57BL/6J WT and Nrf2^-/-^	Not informed	Nrf2^-/-^	SD for 4 weeks.	Nrf2 depletion.	Ashino et al. (2013)
		↑ neointimal formation.			

Male C57BL/6J WT and Nrf2^-/-^	Not informed	Nrf2^-/-^	SD.	Femoral artery injury WT	Ashino et al. (2016)
		↑ neointimal formation after femoral injury.		↑ Nrf2.	
				↓ Keap1.	


*↔, not altered; ↓, decreased; ↑, increased; Adipocyte-specific Nrf2-KO, mice with Nrf2 deletion only in adipose tissue; ApoE^-/-^, knockout mice to apolipoprotein E; BM, bone marrow; cLDL, cholesterol fraction of the Low density lipoprotein; E-25 and E-75, treatment with Epigallocatechin-3-gallate 25 and 75 mg/kg, respectively; Gpx-1, glutathione peroxidase 1; HFD, high fat diet; HFHS + SFN, HFHS diet-fed mice that receive SFN treatment; HFHS, high fat high sugar diet; SFN, sulforaphane; HO-1, Hemeoxygenase 1; Keap1-hypo, hypomorphic Keap1 allele mice; LDL, low density lipoprotein; LDLr^-/-^, knockout mice to low density lipoprotein receptor; Lep^*ob/ob*^ or ob/ob, leptin-deficient mice; LFD, low fat diet; NQO-1, NAD(P)H:Quinone Oxidoreductase 1; Nrf2, Nuclear factor-like 2; Nrf2^-/-^:ob/ob mice, Nrf2 knockout and leptin-deficient mice; Nrf2-KO or NRF2^-/-^, knockout mice to Nrf2; oxPAPC, oxidized 1-palmitoyl-2-arachidonoyl-sn-glycero-phosphocholine; SD, standard diet; WT, wild type mice.*

The Nrf2/Keap1/ARE signaling pathway also represents a mechanism by which the gut microbiome activates a wide range of host signaling and homeostatic processes (reviewed in Murota et al., 2018). The intestinal microbiome and its metabolites display a pivotal role in host physiological processes including immune, metabolic, neurological, and nutritional homeostasis (reviewed in Lynch and Pedersen, 2016). Many of these physiological processes are under influence of ROS generation in the gut epithelia. Alterations in the gut microbiota have recently emerged as major triggers of abnormalities in the integrity of the intestinal barrier, facilitating blood translocation of bacteria and uremic toxins, systemic inflammation and adverse outcomes in obesity and diabetes (reviewed in Belizário et al., 2018). Nrf2 activation in intestinal barrier leads to upregulation of antioxidant enzymes, thereby strengthening the cell’s ability to neutralize several types of free radicals (Zheng et al., 2018). In addition, the relationship between bacterial-dependent ROS generation and Nrf2 pathway activity was recently revealed by observations that lactobacilli-induced and Nox1-mediated generation of ROS evokes Nrf2-dependent activation of cytoprotective antioxidants genes (Jones et al., 2015). In fact, microbe-induced ROS generation and activation of Keap1/Nrf2/ ARE signaling may contribute to our understanding on the mechanisms involved in the genesis of obesity and diabetes, comorbidities associated with increased cardiovascular risk.

## Nrf2 and Type 2 Diabetes Mellitus

There is abundant evidence that oxidative damage caused by free radicals contributes to the pathogenesis and progression of type 2 diabetes mellitus and its complications (reviewed in Dandona et al., 1996; Brownlee, 2001 and Giacco and Brownlee, 2010). Only recently, however, has the role of the Nrf2/Keap-1/ARE pathway in the pathophysiology of this condition and the wide range of its complications, such as diabetic nephropathy and impaired cutaneous wound healing begun to be elucidated (Jiménez-Osorio et al., 2014; reviewed in Uruno et al., 2015; Long et al., 2016). Furthermore, as noted in an excellent and recent review on Nrf2 (David et al., 2017), this pathway is implicated in diabetic damage to the pancreas (Yagishita et al., 2014) and heart (Li et al., 2009). There are promising results provided by animal studies and clinical trials suggesting that activation of this pathway can delay or even reverse type 2 diabetes mellitus-associated dysfunctions (Ichikawa et al., 2009; Uruno et al., 2013).

A consistent alteration in diabetic patients is the presence of endothelial dysfunction, which precedes the development of diabetes-associated vascular complications and may explain, in part, the increased cardiovascular risk in this condition. Endothelial dysfunction in diabetes is associated with enhanced vascular contractility, oxidative stress and vascular inflammation (Zakkar et al., 2009; Tabit et al., 2010 and Hamilton and Watts, 2013). The importance of Nrf2 and its downstream elements in the control of vascular function in diabetes has become increasingly apparent and is reinforced by multiple studies using many of the same agents for protection from conditions other than diabetes. Bardoxolone methyl, a synthetic small molecule activator of the Nrf2/Keap-1 pathway, improves structural and functional changes in animal models of renal disease (Wu et al., 2011; Chin et al., 2013). Its therapeutic potential was extended to clinical trials in type 2 diabetic patients with chronic kidney disease (Pergola et al., 2011). The recent studies published by Tan and Sharma’s group demonstrated that the bardoxolone analog, dh404, at low doses, attenuates atherosclerosis in diabetic Apolipoprotein E (ApoE) knockout mice (Tan et al., 2014) and protects against diabetes-induced endothelial dysfunction, both *in vivo* and *in vitro* (Sharma et al., 2017). Considering that endothelial dysfunction, which is the first step in the development of vascular complications in diabetes, is accompanied by pro-oxidative and pro-inflammatory processes, the atheroprotective effects of dh404 could be mediated by improvement of the endothelial function. In agreement with these observations, increased Nrf2 activity induced, for example, by bardoxolone or sulforaphane, abrogates augmented vascular contraction (Alves-Lopes et al., 2016; Sharma et al., 2017) and attenuates reduced vasodilation in diabetic mice (Lu et al., 2017).

One proposed mechanism for the improvement of endothelial function following Nrf2 activation is the increased expression of a subunit of the BK (big potassium or large conductance, Ca^2+^-activated potassium) channel, the BK-β1, and its attenuated degradation (Lu et al., 2017), a process commonly accelerated by diabetes-induced oxidative stress (Li et al., 2017). Other mechanisms include reduced systemic and vascular oxidative stress as well as increased nitric oxide (NO) bioavailability (Liu et al., 2016; Pereira et al., 2017). Beyond this immediate homeostatic response, long-term consequences of Nrf2 activity have also been described as important culprits of micro-and macrovascular complications associated with diabetes. Accordingly, the literature describe functional connections between Nrf2 and signaling pathways involving nuclear factor-κB (NF-κB), p53, ERK5, mTOR46, heat shock proteins, activator protein-1 (AP-1) and Notch homolog 1, translocation-associated (Drosophila) (NOTCH1) (Hwang et al., 2017). This implies that Nrf2 modulates many cellular activities, beyond its immediate homeostatic and cytoprotective actions, influencing processes as diverse as inflammation, proliferation, apoptosis, cell differentiation, tissue regeneration and even metabolism. In fact, decreased activation of Nrf2 is observed in experimental diabetic cardiomyopathy, along with a decrease in the downstream activity of antioxidant enzymes and increased oxidative stress (Wang et al., 2017). In this sense, activation of the Nrf2 system attenuates vascular remodeling by decreasing proliferation, migration, and fibrotic processes. These effects are mediated by reduced metalloproteinase activity and decreased protein expression of adhesion molecules and TNF-α (Wang et al., 2014; Choi et al., 2015).

Such widespread protective effects of Nrf2 might constitute the underlying mechanism involved in the progression of diabetes-associated complications. These results have also provided strong support for the development of new potent enhancers of Nrf2 activity for the prevention and treatment of many diseases in which both inflammatory and oxidative processes have a key pathogenic role.

## Nrf2 and Atherosclerosis

Atherosclerosis, a progressing inflammatory disease produced by many risk factors, such as diabetes, hypertension, and hyperlipidemia, is one of the major cardiovascular diseases, which, together with myocardial infarction and coronary heart disease, will account for more than 20 million deaths in 2030 (reviewed in Yahagi et al., 2016). Even though much is known about the mechanisms that result in the formation of atherosclerotic plaque, the processes are not entirely understood.

During the atherogenesis process, the build-up of lipids in the arterial intima triggers several changes in the microenvironment of the arterial wall, such as the formation of fatty streaks, endothelial dysfunction, recruitment and activation of immune cells and VSMCs proliferation (reviewed in Raggi et al., 2018). Recruitment and retention of inflammatory cells lead to persistent production of cytokines and ROS that contribute to the progression of atherosclerotic lesion (reviewed in Koelwyn et al., 2018). Increased ROS induces the oxidation of low-density lipoprotein (LDL) to ox-LDL that contributes to oxidative stress and foam cell formation in the arterial wall, aggravating the atherosclerotic plaque formation (reviewed in Koelwyn et al., 2018). In this scenario, the transcription factor Nrf2 is considered a protective signaling molecule, since it induces the expression of many antioxidant genes that may attenuate atherosclerosis progression (Jyrkkänen et al., 2008; reviewed in Guerrero-Hue et al., 2017). For instance, deficiency of GPX-1, a Nrf2 target gene, in mice increases ox-LDL-induced foam cell formation and leads to amplified proliferative activity of peritoneal macrophages, indicating that this gene is atheroprotective (Cheng et al., 2013). Moreover, atherosclerotic lesion development and oxidative stress are accelerated in HO-1 deficient ApoE knockout mice (Yet et al., 2003). Deletion of HO-1 in macrophages increases lipid build-up and foam cell formation and, consequently, the production of ROS and pro-inflammatory cytokines (Orozco et al., 2007). Cheng et al. found that HO-1 expression is increased in vulnerable plaque from patients with symptomatic carotid artery disease and this increase correlates with unstable plaque phenotype. These findings indicate that HO-1 is a major regulator of advanced atherosclerotic lesion progression (Cheng et al., 2009). However, it is not clear whether the induction of HO-1 is a compensatory atheroprotective response, trying to reduce increased levels of ROS in the plaque, or if it contributes to increased plaque vulnerability. Therefore, more studies are necessary to understand the role of HO-1 in advanced atherosclerotic plaque stage.

On the other hand, Nrf2 gene deletion in ApoE knockout mice decreases atherosclerotic lesions at a late stage, whereas it does not affect atherosclerotic lesions in earlier stages (Harada et al., 2012). These observations suggest that Nrf2 inhibition may be atheroprotective in advanced plaques. Additionally, Ishii et al. observed that oxidized lipids induce Nrf2-dependent CD36 scavenger receptor expression in macrophages, resulting in intracellular accumulation of ox-LDL (Ishii et al., 2004). However, deletion of Nrf2 in myeloid cells of LDL receptor knockout mice (LDLr^-/-^) exacerbates atherosclerotic lesions and increases pro-inflammatory genes expression (Collins et al., 2012), indicating that Nrf2 activity in myeloid cells and macrophages modulates the pro-inflammatory vascular milieu associated with atherosclerosis. These conflicting findings demonstrate that Nrf2 may also exhibit pro-atherogenic functions, depending on atherosclerotic lesion stage or animal model.

The accumulation of lipids into the vascular intima is related to oxidative and pro-inflammatory stress that result in endothelial cells dysfunction (reviewed in Sitia et al., 2010). Pro-inflammatory cytokines contribute to monocytes recruitment into the intima by inducing expression of endothelial adhesion molecules and chemokines. Nrf2 activity reduces the inflammatory response in endothelial cells. Ox-LDL-induced expression of vascular cellular adhesion molecule-1 (VCAM-1) and intracellular adhesion molecule-1 (ICAM-1) is reduced by a HO-1 inducer (Zhang et al., 2013). Furthermore, Nrf2/ARE pathway suppresses TNF-α-induced expression of redox-sensitive inflammatory genes, including monocyte chemoattractant protein (MCP)-1 and VCAM-1 (Chen et al., 2006). These results indicate that Nrf2 activation is atheroprotective due to its antioxidant and anti-inflammatory actions that limit the deleterious effects imposed by hyperlipidemic and inflammatory processes to endothelial cells.

Nrf2 anti-atherogenic effects have also been linked to its modulatory effects on migration and proliferation of VSMCs. PDGF-induced VSMCs migration is increased by Nrf2 depletion, and Nrf2-deficient mice exhibit higher neointimal hyperplasia, as shown in a wire injury model (Ashino et al., 2013). Nrf2 control of neointima hyperplasia may be linked to the ability of Nrf2/Keap-1 system to regulate VSMCs apoptosis and, consequently, to inhibit neointimal hyperplasia after vascular injury (Ashino et al., 2016). Also, increased expression of Nrf2 target genes reduces VSMCs proliferation (Duckers et al., 2001; Kim et al., 2009). Moreover, Nrf2 activity is important to maintain VSMCs phenotype (Xu M. et al., 2015) and the activation of Keap-1/Nrf2/NQO1 pathway attenuates VSMCs calcification circulating calciprotein particles (CPP)-induced VSMCs calcification (Aghagolzadeh et al., 2017). Taken together, these studies indicate that Nrf2 may protect against atherogenesis by decreasing VSMCs migration, proliferation, calcification and vascular remodeling.

In recent years, microRNAs (non-coding small RNAs) were identified as key regulators in the cellular events and molecular signaling pathways involved in atherosclerosis (reviewed in Feinberg and Moore, 2016). Multiple microRNAs that participate in cholesterol homeostasis (miR-33), macrophage activation (miR-155), endothelium dysfunction (miR-146), VSMCs proliferation (miR-221), and other processes that lead to plaque progression have already been identified (reviewed in Feinberg and Moore, 2016). In this context, the Nrf2 system and microRNAs can establish regulatory loops and influence vascular responses to oxidative and inflammatory injury. Accordingly, Nrf2 and miR93 regulate endothelial glycolysis, proliferation, and quiescence by Krüppel-like Factor 2 (KLF2)- and vascular endothelial growth factor A (VEGFA)-dependent mechanisms (Kuosmanen et al., 2017). Moreover, oxidized palmitoyl-arachidonoyl-phosphatidylcholine (Ox-PAPC)- induced HO-1 expression is partially dependent on miR-320a in endothelial cells (Schrottmaier et al., 2014). However, the role of microRNAs in the Nrf2 system and their implications in atherogenesis need to be further explored.

In conclusion, anti-oxidant and anti-inflammatory effects of Nrf2 play an essential modulatory role in the formation and progression of atherosclerotic lesions, regulating functional and structural vascular responses. However, additional studies are necessary to explain the discrepant results related to the role of Nrf2 in the different stages of plaque progression. The interactions between microRNAs and Nrf2 target genes during atherosclerosis development also deserve further investigation.

## Conclusion

**FIGURE 1:**
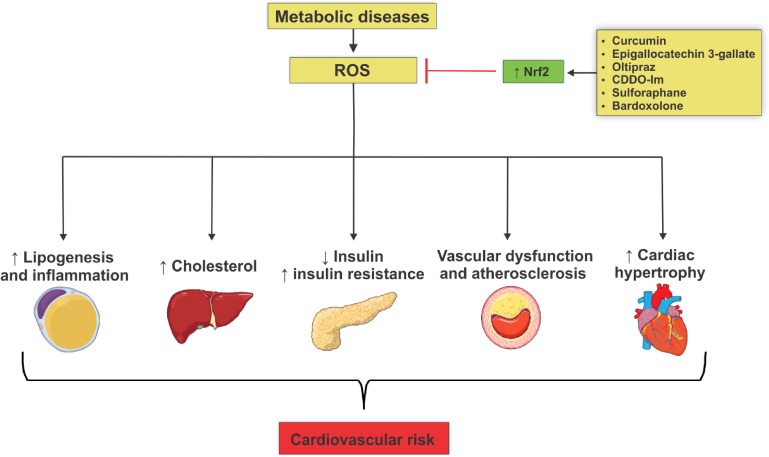
Mechanisms involved in the actions of reactive oxygen species that lead to metabolic diseases and cardiovascular risk development. Metabolic diseases are closely associated with increased generation of reactive oxygen species (ROS) due to reduced Nrf2 antioxidant activity. This phenomenon culminates in target-organ damage and metabolism disorders, such as adipogenesis and adipose tissue inflammation, increased production of hepatic cholesterol, decreased insulin secretion, insulin resistance, loss of integrity of vascular tone control, endothelial dysfunction and atheroma formation, all contributing to increased cardiovascular risk. Nrf2 activation by several agents reduces ROS levels, decreasing metabolic damage and reducing cardiovascular risk.

Activation of the Nrf2-dependent antioxidant system plays an important role in cell defense against oxidative stress damage, whereas the insufficiency of the Nrf2 system is associated with multiple aspects of the genesis and progression of metabolic diseases, posing a great risk to the cardiovascular system (Figure 1). The systemic increase of Nrf2 activity by several activators may be beneficial in the treatment of metabolic diseases. In addition, selective upregulation of Nrf2 genes may represent a potential therapy in obesity, diabetes and atherosclerosis. Looking to the future, experimental research that elucidates the role of Nrf2 activation in specific tissues, such as adipose tissue, liver, pancreas and others, is important for better understanding of the multiple roles of Nrf2. Additional studies may also provide new redox balance-targeted therapy for the treatment of metabolic diseases and consequent mitigation of cardiovascular risk.

## Author Contributions

RdC, DR, CP, JS, JA, NL, and RT equally contributed to the conception and draft of manuscript and approved its final version.

## Conflict of Interest Statement

The authors declare that the research was conducted in the absence of any commercial or financial relationships that could be construed as a potential conflict of interest.
